# Antitumor Effects of L-citrulline on Hela Cervical Cancer Cell Lines

**DOI:** 10.2174/1871520622666220426101409

**Published:** 2022-09-14

**Authors:** Ceren Yildiz Eren, Hulusi Goktug Gurer, Ozlem Ozgur Gursoy, Canan Vejselova Sezer

**Affiliations:** ^1^ Eskisehir Acibadem Hospital, Obstetrics and Gynecology Clinic, Eskisehir, Turkey; ^2^Department of Biology, Faculty of Science, Eskişehir Technical University, Eskisehir, Turkey

**Keywords:** L-citrulline, HeLa, anticancer effects, MTT, Annexin-V, migration assay

## Abstract

**Aim:**

Cervical cancer is the deadliest gynecological malignancy. This study aims to examine the anticancer effects of L-citrulline on HeLa cell culture.

**Materials and Methods:**

HeLa cells were cultured in complete Eagle's minimum essential medium. HeLa cells were seeded in 96-well plates and incubated with L-citrulline. After incubation, MTT dye was added and incubated. Annexin-V technique was used to test the apoptosis. The activated caspases of HeLa cells by L-citrulline exposure were measured with the Caspase 3/7 technique. One-way variance analysis was conducted for statistical analysis by using GraphPad Prism 6.0 for Windows.

**Results:**

L-citrulline showed its toxicity on HeLa cells in a dose-dependent manner in application times of 24 and 48 hours. The IC_50_ dose of L-citrulline was 0.19 and 0.16 mg/mL at 24 and 48 hours, respectively. When HeLa cells were exposed to an IC_50_ dose of L-citrulline for 24 hours, the percentages of the dead, early apoptotic, and late apoptotic cells were detected to be 0.75%, 23.05%, and 12.75%, respectively. The differences in the wideness of the scratch area were observed at the initial stage and after 24 hours of applying L-citrulline.

**Conclusion:**

L-citrulline showed its toxicity on HeLa cells in a dose-dependent manner. Based on Annexin and Caspase findings, it can be concluded that L-citrulline exerted a pro-apoptotic effect on HeLa cells in only a short exposure time. L-citrulline also showed a migration inhibitory effect. The findings of this study indicate L-citrulline to be worthy of investigation for its anticancer activities *in vitro* and *in vivo*, and as a candidate for cancer therapy.

## 1 INTRODUCTION

Cervical cancer is one of the most common causes of death among gynecological cancers. The treatment of early-stage disease includes radical hysterectomy. Chemotherapy and radiotherapy are used in post-hysterectomy treatment [1, 2]. It is estimated that cervical cancer affects approximately 605,000 women each year, of which approximately 341,000 die all over the world [3]. Human papillomavirus infection is the most important risk factor for cervical cancer [4].

*In vitro* cell culture method is used to study the behavior of cells in a controlled environment. Pharmaceutical studies have an important place in that the physicochemical properties of cell cultures, such as pH, temperature, and pressure, can be controlled [5, 6].

MTT is a colorimetric assay used to measure cell viability and cytotoxicity. It is based on reducing the tetrazolium salt *(3-(4,5-dimethylthiazol-2-yl)-2,5-diphenyltetrazolium bromide)* to purple formazan crystals. This reaction is carried out by metabolically active cells containing NADPH-dependent oxidoreductase enzymes [7]. MTT assay is a preliminary assay to assess cytotoxicity. Studies have revealed limitations of the MTT assay and confounding factors. However, the MTT assay is still commonly used [8].

Phosphatidylserine present in the inner plasma membrane is released into the outer layer of the plasma membrane during apoptosis. It is bound by Annexin V (a calcium-binding protein) in the outer layer. Utilizing this mechanism, fluorescently labeled Annexin V can be used for apoptosis detection. Caspases are a large family of cysteine proteases. Caspase-3 plays the most crucial role in the family. The family is required to initiate and execute apoptosis [9]. Mobility is an essential feature of living cells. It is involved in processes, such as cancer metastasis and inflammation. It is essential in cancer research as it demonstrates metastatic progression [10].

L-citrulline is an organic compound and a non-essential amino acid. It can be synthesized endogenously in the body. It is not one of the 20 primary amino acids encoded by DNA, and therefore, is not involved in protein synthesis. L-citrulline is an amino acid made from L-ornithine and carbamoyl phosphate in the urea cycle. L-citrulline is also produced from L-arginine as a by-product. L-citrulline is converted to L-arginine by argininosuccinate synthase. L-arginine is responsible for the therapeutic effects of L-citrulline. L-arginine realizes many of its effects by converting to nitric oxide. Watermelon is the primary source of this amino acid in the diet. The name of the amino acid comes from the scientific name of watermelon, *Citrullus lanatus*. The concentration of L-citrulline in watermelon varies depending on the variety and type. L-citrulline is used to prevent endothelial dysfunction in cardiovascular diseases and diabetes, and treat erectile dysfunction. In addition, due to its role in arginine metabolism, L-citrulline is a potential agent for treating cancer [11].

L-citrulline is converted to L-arginine. L-arginine is the precursor of nitric oxide. Nitric oxide is the precursor of polyamine biosynthesis. Mechanisms of nitric oxide and polyamines in tumor progression are not precise. High levels of nitrous oxide increase oxidative reactions that result in apoptosis. This may be the mechanism of the anticancer effect of nitrous oxide [11]. The relationship between arginine deprivation and inhibition of peptidyl arginine deiminases and cell death has been investigated earlier [12]. This study aims to examine the anticancer effects of L-citrulline in a HeLa cell culture medium.

## 2 MATERIALS AND METHODS

Human cervix adenocarcinoma HeLa (ATCC^®^ CCL-2™) cells were obtained from the American Type Culture Collection (Manassas, USA). L-citrulline was obtained from Akcan Kimya (Turkey); fetal bovine serum (FBS), penicillin/streptomycin, dimethyl sulfoxide (DMSO), 3-(4,5-dimethylthiazol-2-yl)-2,5 diphenyl-2H-tetrazolium bromide (MTT) and Eagle's minimum essential medium (EMEM) were purchased from Sigma-Aldrich (St. Louis, USA). Caspase 3/7 and Annexin-V Kits were from Merck, Millipore, USA. All analyses were repeated three times, and the average of the obtained values was accepted as the analysis result.

### 2.1 Culture

HeLa cells were cultured in a complete EMEM medium containing 100 units/mL-100 μg/mL of penicillin-streptomycin and 10% fetal bovine serum at 37 °C and a 5% CO_2_ incubator. HeLa cells with confluency of 85% were trypsinized for 2 days and used for experimentations [13].

### 2.2 MTT Assay

L-citrulline was diluted in DMSO, and further dilutions of L-citrulline were prepared with a complete EMEM medium. HeLa cells were seeded (5×103/well) in 96 healthy plates and incubated for 24 and 48 hours at 37°C and 5% CO2 with the L-citrulline concentrations. At the end of exposure time, MTT (5 mg/mL) dye was added (20 μL/well) and incubated further for 3 hours. After the incubation, liquids were aspirated from the plates, and DMSO (200 μL/well) was added [14]. Plates were read at 570 nm (n = 3) on an ELISA reader (HTX Synergy, BioTek, USA). Obtained absorbances were used for detecting the viability percentages and IC_50_ concentrations.

### 2.3 Annexin-V Assay

Annexin-V technique was used to test the apoptosis-promoting ability of L-citrulline on HeLa cells. In brief, HeLa cells were seeded in six-well plates (5x10^5^cells/well) and treated with IC_50_ concentration of L-citrulline for 24 hours at 37 °C and 5% CO_2_ conditions. After trypsinization, HeLa cells were washed in phosphate buffer (PBS). 100 μL/sample of Annexin-V solution was added to all samples and incubated for 15 minutes in dark at room temperature (Muse^®^ Annexin-V and dead cell assay kit) [15]. HeLa cells were analyzed with Muse™ cell analyzer (Merck, Millipore, Hayward, California, USA).

### 2.4 Caspase 3/7 Analysis

The activated caspases of HeLa cells by L-citrulline exposure were measured with the Caspase 3/7 technique. For this manner, HeLa cells were exposed to an IC_50_ dose of L-citrulline for 24 hours in six-well plates, and untreated HeLa cells cultured in the same conditions at a density of 5x10^5^/well were harvested by trypsinization and washed in phosphate-buffered saline (PBS). Caspase 3/7 working solution and 7-ADD solutions were added to all samples according to the manufacturer's user manual of the Caspase 3/7 kit (Merck, Millipore, Hayward, California, USA). Samples were read on a cell analyzer (Muse TM Cell Analyzer, Merck, Millipore, Hayward, California, USA).

### 2.5 Migration Assay

HeLa cells (3 × 10^5^ cells/well) were plated in 6-well plates and incubated for 24 hours. Monolayer confluent cells on the plates were scratched vertically with a sterile 20–200 μL pipette tip and washed with phosphate-buffered saline (PBS). 3 mL of fresh EMEM and medium containing L-citrulline were added to the wells and imaged for the initial view of the artificial wound at the 0^th^ hour. The scratch closure was imaged after 24 hours of incubation under a light microscope. Untreated cells were used as a control group [16].

### 2.6 Statistical Analysis

One-way variance analysis for multiple comparisons was employed to analyze the results using GraphPad Prism 6.0 for Windows.

## 3 RESULTS

### 3.1 MTT Findings

**Fig. (1):**
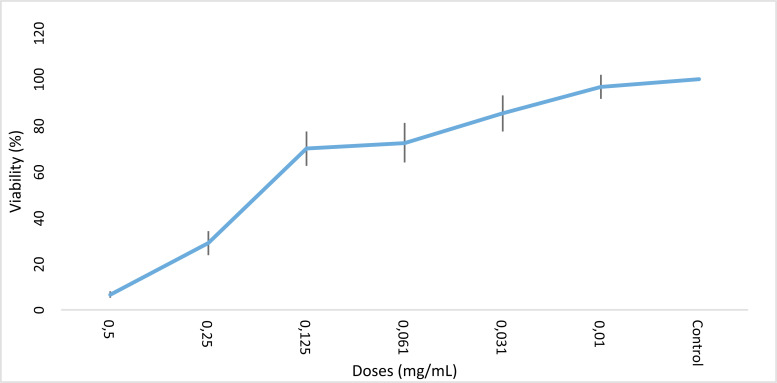
Growth inhibition activity of L-citrulline on HeLa cells. IC_50_ dose of L-citrulline 0.19 mg/mL for 24 hours (*: *p*<0.5).

**Fig. (2):**
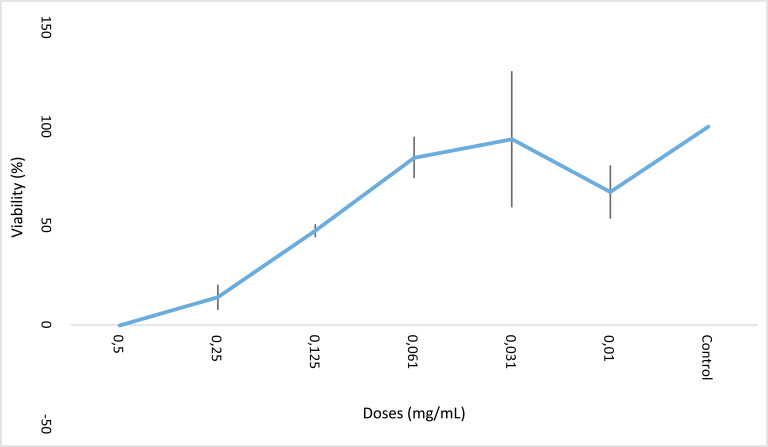
Growth inhibition activity of L-citrulline on HeLa cells. IC_50_ dose of L-citrulline 0.16 mg/mL for 48 hours (*: *p*<0.5).

L-citrulline showed its toxicity on HeLa cells in a dose-dependent manner in application times of 24 and 48 hours (Figs. **1** and **2**). The highest decrease was detected at 0.5 mg/mL of L-citrulline at 24 and 48 hours of exposure. IC_50_ values of L-citrulline were detected to be 0.19 mg/mL and 0.16 mg/mL for 24 and 48 hours of exposure, respectively. Furthermore, the recorded data imply the efficiency of L-citrulline for growth inhibition and cytotoxicity in both the application periods. At 24 and 48 hours of exposure, each value was found to be significant statistically concerning the control.

### 3.2 Annexin-V Findings

**Fig. (3):**
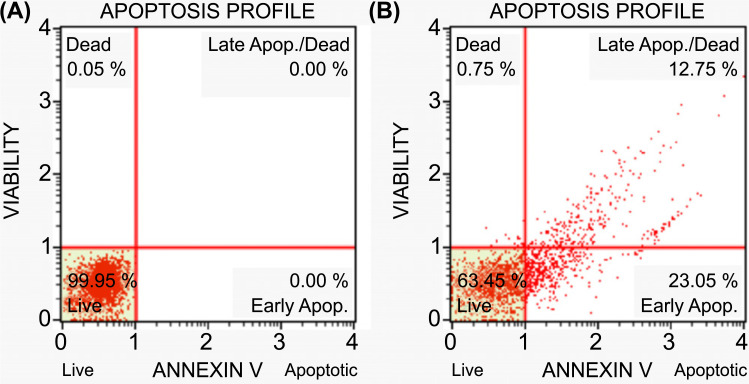
Apoptosis profiles of HeLa cells. **A**) Control HeLa cells; Live cells 99.95% were detected. Percentages of late apoptotic, early apoptotic and dead cells were detected as 0.00%. **B**) HeLa cells were exposed to an IC50 dose of L-citrulline for 24 hours. 63.45% of cells were live. The percentages of the dead, early apoptotic and late apoptotic cells were detected to be 0.75%, 23.05% and 12.75%, respectively.

**Fig. (4):**
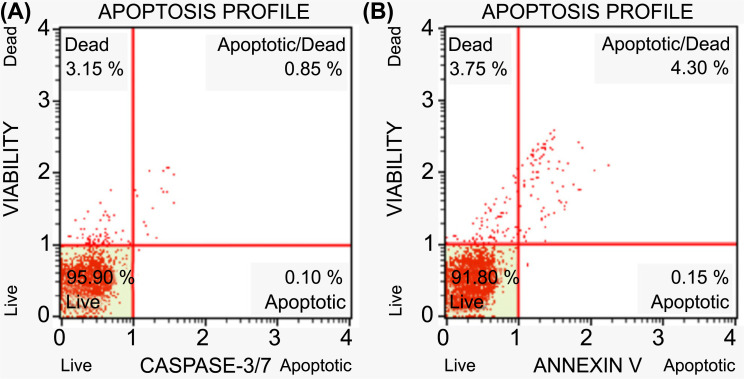
Caspase-dependent apoptosis profiles of *HeLa* cells. **A**) Untreated HeLa cells (95.90% live, 3.15% dead, 0.85 apoptotic/dead, and 0.10% apoptotic cells). **B**). HeLa cells treated with IC50 concentration of L-citrulline for 24 hours (91.80% live, 3.75% dead, 4.30% apoptotic/dead, and 0.15% apoptotic cells).

These profiles of HeLa cells showed that L-citrulline promoted apoptosis (Figs. **3A**, **B** and **4A**, **B**). Compared to HeLa control cells, L-citrulline triggered apoptosis in the treated HeLa cells at 35.80% (Fig. **3B**). Based on this finding, it can be concluded that L-citrulline is proapoptotic on HeLa cells in a short time of exposure.

### 3.3 Caspase 3/7 Results

#### 3.3.1 Migration Assay Findings

The differences in the wideness of the scratch area were observed at the initial stage and after 24 hours of applying L-citrulline. It was shown that the precise area of the artificial wound at the start of incubation was closed totally with the proliferated HeLa cells after incubation of 24 hours.

**Fig. (5):**
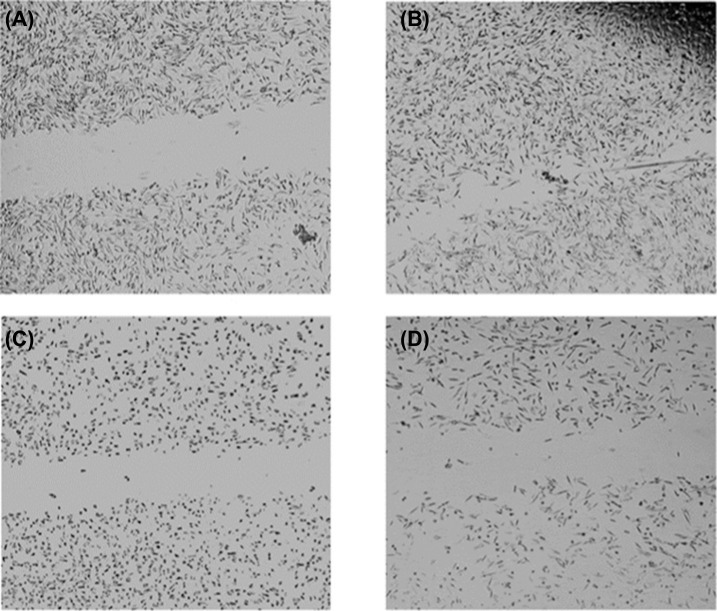
Migration capability of HeLa cells. **A**) Control HeLa cells at the 0th hour. **B**) Control HeLa cells after 24 hours. **C**) HeLa cells treated with L-citrulline at the 0^th^ hour. **D**). HeLa cells treated with L-citrulline at the 24th hour.

**Fig. (6):**
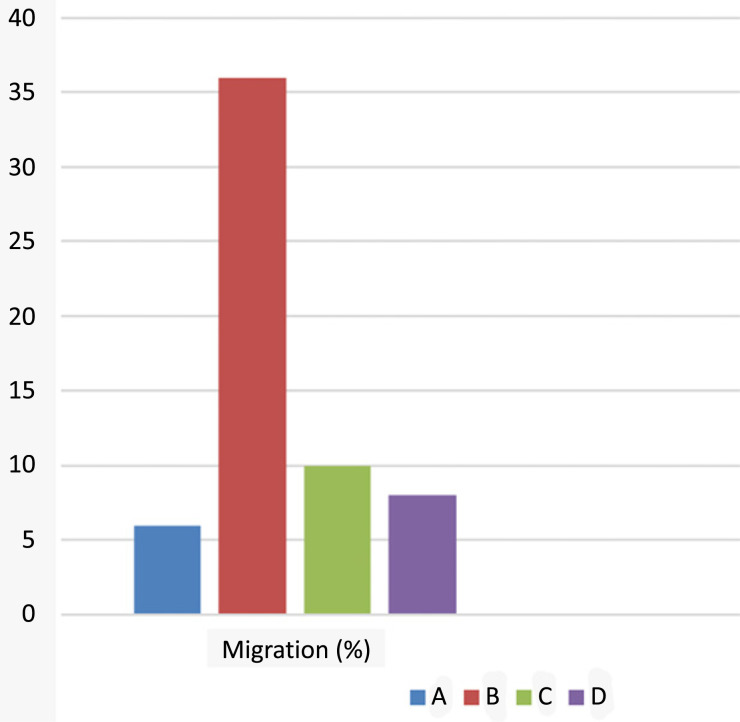
Migration percentage of HeLa cells. **A**) Control HeLa cells at the 0th hour. **B**) Control HeLa cells after 24 hours. **C**) HeLa cells treated with L-citrulline at 0^th^ hour. **D**) HeLa cells treated with L-citrulline at 24^th^ hour.

This finding implies the migration ability of HeLa cells. In the HeLa cells exposed to IC_50_ concentration of L-citrulline for 24 hours, the scratch area was not closed. This was evaluated as the migration inhibitory effect of L-citrulline at a concentration of 0.19 mg/mL in an application period of 24 hours. This finding also points to the antiproliferative and cytotoxic effects of L-citrulline on HeLa cells (Figs. **5A**-**D** and **6A**-**D**).

## 4 DISCUSSION

According to the data obtained in our study, L-citrulline significantly inhibited the proliferation of HeLa cancer cells in a dose-dependent manner at 24 and 48 hours of administration (*p*<0.05). Considering the apoptosis profiles of HeLa cells, it was determined that 63.45% of HeLa cells exposed to IC_50_ L-citrulline for 24 hours were alive. The percentages of the dead, early apoptotic, and late apoptotic cells were determined as 0.75%, 23.05%, and 12.75%, respectively. These profiles of HeLa cells suggest that L-citrulline promotes apoptosis. L-citrulline showed an anti-migration effect after a 24-hour application.

L-citrulline is a neutral, non-essential alpha-amino acid that is an essential component of the urea cycle in the liver and kidneys. As a non-protein amino acid, it is rarely found in food but is highly concentrated in watermelon. There is increasing evidence that endothelial dysfunction has its origins in inadequate L-arginine-nitric oxide metabolism. The effect of L-arginine supplementation on nitric oxide metabolism is negligible. This has accelerated studies exploring the potential therapeutic benefits of L-citrulline. Like many other nitric oxide-boosting supplements, L-citrulline is gaining attention for its potential cardiovascular and anti-hypertensive abilities [17, 18].

L-citrulline increases arginine synthesis and indirectly increases nitric oxide biosynthesis. This leads to an increase in some functions, especially the endothelial vasodilating function. These studies in various animal models have demonstrated the critical role of nitric oxide in endothelial protection against atherogenic dietary conditions. L-citrulline effectively promotes increased endogenous nitric oxide production and reduces the detrimental effect of oxidative stress on nitric oxide. In addition to these, L-citrulline has also been studied for its properties, such as increased mitochondrial oxidative capacity [19].

In the light of data obtained from many studies, it is known that high nitric oxide levels obtained from nitric oxide donors inhibit the proliferation of tumor cells and induce apoptosis of tumor cells [20]. In addition, it has been shown that high nitric oxide levels can sensitize resistant tumor cells to treatment [21]. It has been determined that nitric oxide has selective inhibitory effects on the growth of tumor cells compared to normal cells and that it is very effective against cancer cells that show resistance to drugs by overexpressing some protein structures that program drug resistance [22].

The discovery of nitric oxide-mediated pathways in cancer has paved the way for new nitric oxide-based therapies. Some of these new treatments involve direct delivery of nitric oxide to the tumor. Such increases in nitric oxide reverse chemotherapeutic and radiotherapeutic resistance mediated by hypoxia-induced transcription factors. Some preclinical research has suggested other mechanisms. Numerous studies support the effectiveness of nitric oxide-releasing biomolecules in aiding nitric oxide release. Many clinical studies show the efficacy of treatments with nitric oxide, either alone or in addition to radio, immuno, and chemotherapy [23-25].

According to clinical studies, drug resistance seen in treatment with anticancer agents may be related to many factors. It has been suggested that controlling key signaling pathways related to resistance mechanisms may be the most effective way to eliminate drug resistance in cancer patients. It has been reported that drug resistance can be prevented in cancer cells by inhibiting the Ras/Raf/MEK/ERK, PI3K/AKT pathways, and key transcription factors, such as NF-κB and HIF-1α [1]. It has been reported that the addition of nitric oxide to anticancer therapy acts by triggering inhibition of HIF-1α expression and phosphorylation of NF-kB, AKT, and ERK. It has been noted that controlling key signaling pathways related to resistance mechanisms using nitric oxide may be the most effective way to eliminate drug resistance in cancer patients [2].

Recent studies have examined some effects of nitric oxide. They reported that the drug resistance of HL60 (human leukemia cell line) cells exposed to a nitric oxide-releasing prodrug (PABA/nitric oxide) for six months was highly reduced, and the sensitivity of the resistant cells returned within three weeks after PABA/nitric oxide was removed [26].

Nitric oxide is an agent with antitumorigenic effects, and the mechanisms of these effects have been investigated. Nitric oxide has an antitumorigenic effect that contributes to macrophage-induced cytotoxicity. It has been reported to have a cytotoxic effect due to the loss of intracellular iron and inhibition of mitochondrial respiration and DNA synthesis in tumor cells. Nitric oxide can modulate tumor metabolism by preventing respiration, changes in mitochondrial mass, inhibition of bioenergetic enzymes, and stimulation of secondary signaling pathways. It inhibits DNA synthesis by inhibiting ribonucleotide reductase, a rate-limiting enzyme in DNA synthesis. Long-term nitric oxide levels increase p53 expression through Caspase family proteases and promote apoptosis by promoting the expression of proapoptotic proteins [27].

Nitric oxide and reactive oxygen species can also kill several cancer cells or inhibit treatment resistance in cancer types, such as melanoma, cervical cancer, and bladder cancer. Mice lacking nitric oxide synthetase, which produces nitric oxide and L-citrulline from L-arginine, developed more rapidly lymphomas and sarcomas. These mice were determined to have a lower apoptotic index. Transfection of structures expressing nitric oxide synthase enzyme into melanoma cells has also been shown to inhibit tumor growth and metastasis [28, 29].

Nitric oxide is biosynthesized endogenously from L-arginine and oxygen by different nitric oxide synthase enzymes and reduction of inorganic nitrate. It has been shown that cells containing nitric oxide synthase can reconvert L-citrulline, a by-product of nitric oxide synthesis, into L-arginine *via* the arginine-citrulline cycle. L-citrulline, which can be converted to L-arginine, is a second nitric oxide donor in the nitric oxide synthase-dependent pathway. This way, increased L-citrulline is converted to nitric oxide and increases nitric oxide synthesis and effects [30].

Our research topic of anticancer effects of L-citrulline is an extremely new and untouched research area. Many effects of L-citrulline have been investigated. Some of these have been mentioned above. From the results obtained, it can be concluded that L-citrulline shows its effects by increasing nitric oxide synthesis. Although many effects of L-citrulline have been investigated, it is challenging to come across studies investigating its anticancer activity. However, the results obtained in our study are quite remarkable. L-citrulline has shown significant anticancer activity on human cervical cancer cell lines. In the literature, it is seen that studies on the subject generally focus on the anticancer effects of nitric oxide, and there are no clinical or experimental studies on the anticancer effects of L-citrulline. Due to this situation, our research results could not be compared with the results of other studies.

In addition to its anticancer effects, L-citrulline also gives hope for drug resistance. The essential drawback of the drugs used in cancer patients today is the drug resistance encountered. Both nitric oxide and L-citrulline promise important developments in this regard. Both the agents can be investigated as a solution against drug resistance. At this point, L-citrulline can be a solution to drug resistance by acting directly through its effects and indirectly by increasing nitric oxide. Today, combined therapy comes to the fore in treating cancer patients, and the treatment is carried out with more than one drug. It is expected that the addition of an agent such as L-citrulline, which prevents drug resistance, to these combinations will bring new perspectives to treatment protocols.

L-arginine treatment may have adverse effects. Therefore, oral L-citrulline is a substitute for L-arginine supply. L-citrulline is essential in supplying L-arginine to NOS by converting it into L-arginine. However, the clinical utility of L-citrulline is limited, and L-citrulline is not very organ-specific [31]. Computational design of analogs of L-citrulline has been reported [32].

No analysis was performed with the “reference standard drug” in our research. This is because cervical cancer is not chemosensitive. A guideline for use in the treatment of cervical cancer has been published by FIGO (Fédération Internationale de Gynécologie et d'Obstétrique). This guideline emphasizes that surgery and radiotherapy are mainly used in the treatment of cervical cancer. This is because cervical cancer is not chemosensitive but radiosensitive [33].

In this regard, the approach of international organizations is to use chemotherapy in cervical cancer only to increase radio sensitivity. For this purpose, platinum group agents are generally preferred. Some studies have determined that chemotherapy has no curative effect in the treatment of cervical cancer [34]. There is no approved chemotherapeutic agent for use alone or in combination for cervical cancer. Therefore, our study examined no agent as a “reference standard drug.”

One of the limitations of our research is that the effects of L-citrulline and nitric oxide on cancer cells were not examined. It is considered that examining the effects and mechanisms of these agents with new studies to be planned will shed light on the subject. The strength of this research is that it is original. Although research on L-citrulline has focused on different areas, studies examining the potential anticancer properties of this agent are minimal. In this respect, it is possible to say that our research is original. Another limitation is that the efficacy of L-citrulline has not been compared with any standard drug.

## CONCLUSION

Based on all experimental findings of this study, it can be concluded that L-citrulline showed its toxicity on HeLa cells in low doses with antiproliferative and caspase-independent proapoptotic effects within the application period of 24 hours. Consequently, L-citrulline is worthy of investigation for its anticancer activities *in vitro* and *in vivo*, and as a candidate for cancer therapy.

## Data Availability

Not applicable.

## LIST OF ABBREVIATIONS

DMSO: Dimethyl Sulfoxide
EMEM: Eagle's Minimum Essential Medium
PBS: Phosphate-Buffered Saline
